# Complete Genome Sequence of a Novel Strain of Infectious Bronchitis Virus, Isolated from Chickens in China in 2016

**DOI:** 10.1128/genomeA.01277-17

**Published:** 2017-11-09

**Authors:** Ting-Ting Li, Jin-Yan Li, Tao Huang, Xing-Yi Ge

**Affiliations:** a Hubei Animal Disease Prevention and Control Center, Wuhan, China; b College of Biology, Hunan University, Changsha, China

## Abstract

An avian infectious bronchitis virus (IBV) was detected from trachea swabs of chickens in Hubei province, China, in 2016. The complete genome of the IBV strain, CK/CH/HB/2016, was characterized and analyzed to better understand IBV epidemiology in China.

## GENOME ANNOUNCEMENT

Avian infectious bronchitis virus (IBV) belongs to the genus *Gammacoronavirus* in the family *Coronaviridae* of the order* Nidovirales* (1). IBV is a highly contagious pathogen transmitted through the respiratory tract and causes infectious bronchitis in chickens, leading to serious economic consequences worldwide (2). Besides chickens as the major host, IBV can also infect ducks, geese, pigeons, and peafowl (1).

To investigate the genetic diversity of IBV, we monitored the intensive farms in Hubei province, China. During 2016, seven batches of chickens from three farms showed typical clinical signs of infectious bronchitis. Out of 127 trachea swab samples, 122 (96.1%) were detected as IBV positive by one-step reverse transcriptase-PCR testing with primers IBVNF (5′ GTTTGARGGTAGYGGYGTYCCTGA 3′) and IBVNR (5′ CAGMACCHTTDRCAGCAACCCACACTA 3′). Subsequently, a complete genome sequence of the IBV strain, CK/CH/HB/2016, was characterized by a previously described method (3, 4).

The complete genome of CK/CH/HB/2016 comprised 27,685 nucleotides (nt), excluding the 3′ poly(A) tail. Genomic alignment showed that CK/CH/HB/2016 shares nt identity percentages of 97.4% and 96.5% with CK/CH/SD09/005 (GenBank accession number KF668605) and GX-NN09032 (GenBank accession number JX897900), respectively (5, 6). Ten open reading frames (ORFs) in the CK/CH/HB/2016 genome were found, including 1a, 1b, S (spike), 3a, 3b, E (envelope), M (membrane), 5a, 5b, and N (nucleocapsid). In its genome, two variant transcription-regulating sequences (TRSs) were found, a highly conserved TRS, CTTAACAA, located ahead of ORFs 1a, 1b, 3a, M, 5a, and N, and a variant TRS, AAGAACAA, located ahead of S. An amino acid deletion, ^169^T, in the N gene of CK/CH/HB/2016 was detected, which is rare for IBVs (7).

The frequency of recombination was one of the most important reasons for the emergence of novel IBV strains (8–11). By recombination analysis, nine potential recombinant events between CK/CH/HB/2016 and previously identified IBV strains were detected by multiple methods, which indicated a complicated origin of the novel CK/CH/HB/2016 strain (12). The hot spots of recombination were found in the nsp2, nsp3, nsp4, nsp5, nsp6, nsp11, nsp12, and N genes.

The coronavirus spike, which is also a highly variable gene, is responsible for virus entry through receptor binding and membrane fusion (13). A sequence comparison revealed that the spike of CK/CH/HB/2016 had 98.2%, 98%, and 72.8% amino acid sequence identities to those of CK/CH/SD09/005, GX-NN09032, and Ck/Aus/N1/88 (GenBank accession number KU556804), respectively. Interestingly, Ck/Aus/N1/88 is an IBV strain that was detected in Australia (11). Phylogenetic analyses based on the full-length spikes and the predicted variable receptor binding domains both showed that CK/CH/HB/2016, CK/CH/SD09/005, GX-NN09032, and two strains detected in Australia, Ck/Aus/N1/88 and Ck/Aus/N1/03 (GenBank accession number KU556806), were clustered together. This result suggests that the spike genes of these IBV strains have the same origin.

### Accession number(s).

The genome sequence of IBV strain CK/CH/HB/2016 was deposited in GenBank under the accession number MF882923.
